# Molecular Simulation Study on the Hydrogen Permeation Behavior and Mechanism of Common Polymers

**DOI:** 10.3390/polym16070953

**Published:** 2024-03-30

**Authors:** Xuemin Zhang, Lizhen Zhai, Houbu Li, Guoquan Qi, Xiong Gao, Wenhui Yang

**Affiliations:** 1 School of Materials Science and Engineering, Chang’an University, Xi’an 710064, China; 2 State Key Laboratory for Performance and Structure Safety of Petroleum Tubular Goods and Equipment Materials, CNPC Tubular Goods Research Institute, Xi’an 710077, China; 3 Shaanxi Yanchang Petroleum Northwest Rubber LLC, Xianyang 712023, China

**Keywords:** hydrogen, permeability, polymers, adsorption, diffusion, molecular simulation

## Abstract

This research aimed to provide an understanding of the selection and safe application of pipeline liner materials for hydrogen transport by examining the permeation properties and mechanisms of hydrogen within polymers commonly used for this purpose, such as high-density polyethylene (HDPE) and ethylene-vinyl alcohol copolymer (EVOH), through molecular simulation. The study was carried out within defined operational parameters of temperature (ranging from room temperature to 80 °C) and pressure (from 2.5 to 10 MPa) that are pertinent to hydrogen pipeline infrastructures. The results reveal that with an increase in temperature from 30 °C to 80 °C, the solubility, diffusion, and permeability coefficients of hydrogen in HDPE increase by 18.7%, 92.9%, and 129.0%, respectively. Similarly, in EVOH, these coefficients experience increments of 15.9%, 81.6%, and 112.7%. Conversely, pressure variations have a negligible effect on permeability in both polymers. HDPE exhibits significantly higher hydrogen permeability compared to EVOH. The unique chain segment configuration of EVOH leads to the formation of robust hydrogen bonds among the hydroxyl groups, thereby impeding the permeation of hydrogen. The process by which hydrogen is adsorbed in polymers involves aggregation at low potential energy levels. During diffusion, the hydrogen molecule primarily vibrates within a limited range, with intermittent occurrences of significant hole-to-hole transitions over larger distances. Hydrogen exhibits a stronger interaction with HDPE compared to EVOH, leading to a higher number of adsorption sites and increased hydrogen adsorption capacity in HDPE. Hydrogen molecules move more actively in HDPE than in EVOH, exhibiting greater hole amplitude and more holes in transition during the diffusion process.

## 1. Introduction

Hydrogen serves as a crucial industrial resource and energy carrier [1] due to its environmentally friendly attributes of cleanliness and zero emissions. It has emerged as a pivotal focus in the realm of energy transition and a key avenue towards achieving carbon neutrality [2]. In the entirety of the hydrogen energy industrial process, the safe and effective transportation of hydrogen emerges as a crucial element that is necessary for enabling widespread accessibility to this energy source. The delivery of hydrogen can be achieved through three primary pathways: gaseous hydrogen delivery, liquid hydrogen delivery, and material-based hydrogen carriers. Each pathway is predominantly contingent upon the storage methodology employed.

In the transportation of gaseous hydrogen, various methods are utilized, such as compressed hydrogen pressure vessels, tube trailers, and gas pipelines. Among these methods, pipelines provide a cost-effective and efficient solution for hydrogen transport due to their high capacity, extensive interconnectivity, wide geographic coverage, and relatively low operational expenses [3]. Currently, there are more than 5000 km of hydrogen pipelines available globally, with around 2600 km situated in the United States [4]. Generally, hydrogen pipelines are predominantly constructed using ferritic stainless steel, austenitic stainless steel, and other materials [5,6,7]. Nevertheless, the phenomenon of hydrogen embrittlement can accelerate the growth of fatigue cracks in pipelines when exposed to a hydrogen-rich environment, ultimately increasing the likelihood of failure in pipeline steel [8,9,10].

One of the approaches to addressing the impact of hydrogen on pipeline steel is to use non-metallic composite pipelines, such as reinforced thermoplastic composite pipes (RTPs). RTPs commonly consist of an inner thermoplastic liner, multiple fiber reinforcement layers, and an outer protective layer [11]. Thermoplastic liners absorb gases under high pressures and temperatures due to the intrinsic characteristics of polymer materials. Gas molecules typically adsorb on the surface of the polymer, then diffuse internally, and eventually penetrate through to the other side of the polymer lining [12]. If rapid depressurization occurs, the built-up gas is unable to disperse through diffusion, leading to the occurrence of blistering [13]. In contrast to other gas molecules, hydrogen molecules exhibit increased mobility within polymers due to their smaller size. Studies have shown that the permeation rate of hydrogen is approximately 4 to 5 times higher than that of methane in common polymer pipelines [14]. Therefore, investigating hydrogen permeation within the thermoplastic liner of reinforced thermoplastic pipes (RTPs) is crucial to ensure the safe and effective conveyance of hydrogen.

Currently, gas permeability research techniques are primarily categorized into experimental approaches and molecular simulation methods. Chen [15] conducted research on the dissolution and diffusion behavior of hydrogen in polyethylene using gravimetry and nuclear magnetic resonance methods. The study revealed that the solubility of hydrogen in polyethylene film increased with temperature (298–363 K). Using the differential pressure method, Zhang [16] studied the hydrogen permeability of HDPE, the material used as the liner in type IV high-pressure hydrogen storage cylinders. The test results showed that the permeability of hydrogen decreased as the crystallinity of the sample increased, while it increased with rising temperatures (288–353 K). Gay [17] determined the hydrogen diffusion properties of HDPE and epoxy resin films. They found that the gas diffusion significantly influenced the permeation process. Siracusa [18] studied the gas transport parameters of six oriented polypropylene (BOPP) films and observed that the gas transport process follows the Arrhenius model. Chen [19] calculated and simulated the dissolution and diffusion behavior of various gases (H_2_, N_2_, O_2_, H_2_O, CO_2_, H_2_S, SO_2_) in butyl rubber. The results showed that the smaller the gas molecule’s diameter, the larger the diffusion coefficient. Nevertheless, the abovementioned scholars did not investigate the effects on the hydrogen permeation process. Zheng [20] conducted a study on the solubility and diffusion properties of hydrogen in amorphous polyethylene at temperatures ranging from 270 to 310 K and pressures between 0.1 and 0.7 MPa. However, the research pressure levels used in their study do not align with the demands of hydrogen pipeline operations.

While several research studies have explored the phenomenon of hydrogen permeation in polymers, there is still a lack of comprehensive understanding regarding the process and mechanism underlying hydrogen transport within these materials. The studied temperature and pressure ranges are also inconsistent with the working conditions of the hydrogen pipeline. In this paper, to investigate hydrogen permeation within the thermoplastic liner of RTPs with a good hydrogen barrier, high-density polyethylene (HDPE), which is the most commonly used liner material, and ethylene-vinyl alcohol copolymer (EVOH), which is a barrier material against other gases and organic substances [21], were selected as the research objects. The adsorption and diffusion behavior of hydrogen in HDPE and EVOH were investigated through molecular simulation at temperatures ranging from 30 to 80 °C and pressures between 2.5 and 10 MPa. Molecular simulation was employed to investigate the adsorption and diffusion behaviors of hydrogen within HDPE and EVOH. This method allows for a detailed examination of the intricate interactions between hydrogen and polymers, addressing the challenges associated with experimental studies on hydrogen permeation. The impact of temperature and pressure on the hydrogen permeation process in polymers was assessed to elucidate the characteristics and mechanisms underlying hydrogen permeation. Through our findings, we aim to offer insights into the selection and safe utilization of hydrogen pipeline liner materials.

## 2. Theoretical Basis of Permeability

The permeability coefficient *P* serves as a measure of the ability of gas molecules to traverse polymer substances. In accordance with the solution-adsorption model, gas permeability depends on its dissolution and diffusion capabilities [22]. The permeability coefficient *P* can be calculated according to Equation (1):

(1) P=S×D

where *P* is the permeability coefficient, cm^3^ (STP)·cm·cm^−2^·s^−1^·Pa^−1^; *S* is the solubility coefficient, cm^3^ (STP)·cm^−3^·Pa^−1^; and *D* is the diffusion coefficient, cm^2^·s^−1^.

The determination of the solubility coefficient (*S*) of gas molecules within a polymer can be achieved through the application of the adsorption isotherm method. Under consistent temperature conditions, the adsorption isotherms are derived based on the respective solubility concentrations corresponding to various fugacities. Henry’s law is relevant to systems characterized by minimal solubility, and it can be utilized to explain the process of small gas molecules like hydrogen dissolving in polymers [23]. This relationship is represented by Equation (2). Notably, as the fugacity tends towards zero, the solubility coefficient can be ascertained as the limiting slope of the adsorption isotherm, as demonstrated in Equation (3):

(2) $$C=K_{H}\cdot f$$

(3) $$S=\underset{f\to 0}{\lim}\frac{C}{f}=K_{H}$$

where *C* is the dissolved concentration of gas molecules in the polymer, cm^3^ (STP)·cm^−3^; *K_H_* is Henry’s constant; *f* is fugacity, Pa; and *S* is the solubility coefficient, cm^3^ (STP)·cm^−3^·Pa^−1^.

The diffusion coefficient (*D*) can be determined through the analysis of the mean square displacement (MSD) of molecular motion. The calculation is based on the assumption that diffusion is limited to the amorphous section of the polymer, and that this amorphous region is consistent throughout. In the context of molecular dynamics (MD) simulation, the correlation between MSD and the duration of molecular motion can be established by tracking the trajectory of the center of the penetrant molecule. Subsequently, the diffusion coefficient is computed using Einstein’s formula [24], as shown in Equation (4):

(4) $$D=\frac{1}{6N}\underset{t\to \infty }{\lim}\frac{d}{dt}\sum_{i=1}^{N}{\left[r_{i}\left(t\right)-r_{i}\left(0\right)\right]}^{2}$$

where *D* is the diffusion coefficient, cm^2^·s^−1^; *N* is the number of molecules; $r_{i}\left(0\right),\;r_{i}\left(t\right)$ are the initial and final positions of the molecular centroid at time interval *t*; and ${\left[r_{i}\left(t\right)-r_{i}\left(0\right)\right]}^{2}$ is the ensemble mean of MSD of the molecule.

Given that the mean square displacement (MSD) value is calculated by averaging over *N* data points, the parameter “*a*” is defined as the gradient of the MSD curve derived from molecular simulation. Equation (4) can be simplified into Equation (5).

(5) D=a/6

## 3. Model Construction and Parameter Setting

### 3.1. Selection of Force Field and Simulation Conditions

The COMPASS force field represents a pioneering molecular force field that is developed through ab initio calculations, enabling precise forecasting of the configuration, conformation, vibrational behavior, and thermodynamic characteristics of both isolated and condensed molecules [25]. This force field applies to diverse systems, encompassing prevalent polymer and inorganic molecules, thus making it the preferred choice for the study discussed in this paper. The total energy encompassed within the polymer system under the COMPASS force field can be determined using Equation (6):

(6) $$E_{total}=E_{bond}+E_{cross}+E_{non-bond}$$

where $E_{bond}$ represents the bond energy, including the valence angle bending energy, bond stretching energy, inversion energy, and dihedral angle torsion energy; $E_{cross}$ is the cross-term energy; the non-bond energy, represented by $E_{non-bond}$, is composed of the van der Waals and electrostatic energies [26].

In this study, considering the operational parameters of the hydrogen pipeline, such as the operating temperature (room temperature to 80 °C) and pressure (2.5 to 10 MPa), simulated temperatures of 30 °C, 40 °C, 60 °C, and 80 °C were selected for analysis. The maximum delivery pressure of the hydrogen pipeline was maintained below 10 MPa, typically regulated not to exceed 7 MPa, with 4 MPa being the predominant delivery pressure utilized. Consequently, pressure values of 2.5 MPa, 4 MPa, 6 MPa, and 10 MPa were specifically chosen for the simulation procedures.

### 3.2. Model Construction

**Figure 1:**
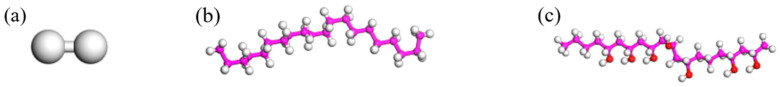
Molecular chain models of gases and polymers: (**a**) H2; (**b**) HDPE; (**c**) EVOH (the white atoms are hydrogen atoms, the purple atoms are carbon atoms, the red atoms are oxygen atoms).

**Figure 2:**
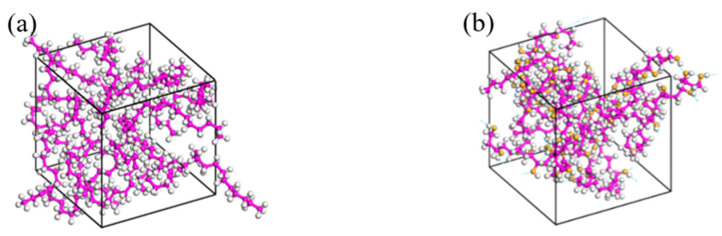
Adsorption cell model: (**a**) HDPE; (**b**) EVOH (the white atoms are hydrogen atoms, the purple atoms are carbon atoms, the yellow atoms are oxygen atoms).

**Figure 3:**
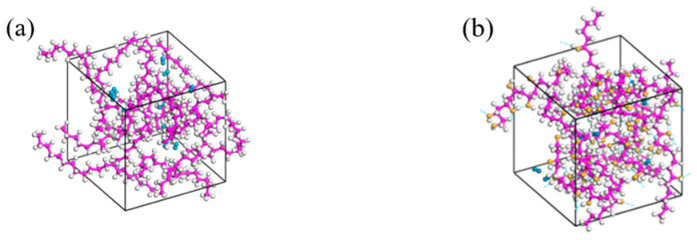
Diffusion cell model: (**a**) H_2_/HDPE; (**b**) H_2_/EVOH (the blue molecules are hydrogen molecules, the white atoms are hydrogen atoms, the purple atoms are carbon atoms, the yellow atoms are oxygen atoms).

In this study, molecular models are developed and molecular dynamics simulations are performed to investigate the dissolution and diffusion behaviors using Materials Studio. The hydrogen molecule and the single-chain models of HDPE and EVOH were built using the Visualize module, as shown in Figure 1. Subsequently, geometric optimization was performed on the hydrogen molecule and polymer chains mentioned above, resulting in energy convergence to 6 × 10^−8^ kcal/mol. This process ensured that the energy level reached a minimum, thereby stabilizing the molecular chains in a structurally stable state. Next, the Amorphous Cell module was employed to generate remotely disordered, short-range ordered unit cells with three-dimensional periodic boundary conditions. Among them, the dimensions of the HDPE and EVOH cells were 17.8 Å × 17.8 Å × 17.8 Å and 19.9 Å × 19.9 Å × 19.9 Å, respectively. As shown in Figure 2, the adsorption cell model comprises 12 HDPE or EVOH stable molecular chains. Likewise, the diffusion cell model (Figure 3) includes 12 hydrogen molecules and 12 stable molecular chains of HDPE or EVOH. Moreover, EVOH contains many hydroxyl groups, leading to significant intermolecular forces, specifically hydrogen bonding, within the material. This results in a more compact arrangement of chain segments in EVOH, contributing to its effective barrier properties.

### 3.3. Relaxation Treatment

**Figure 4:**
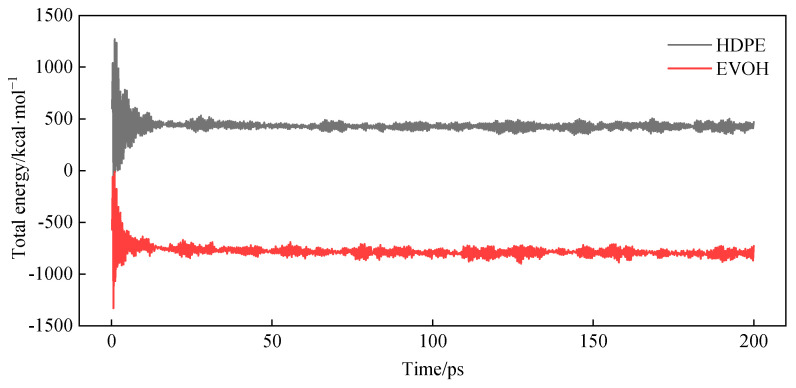
Total energy changes in the cells during dynamic treatment.

The constructed cellular models underwent 25 cycles of annealing within a temperature range of 27 °C to 527 °C (300−800 K), with geometric optimization conducted following each cycle. Subsequently, the configuration exhibiting the lowest energy was chosen and subjected to NVT (constant temperature and constant volume) for 200 ps, followed by NPT (constant temperature and constant pressure) for another 200 ps. The changes in total energy during the dynamic treatment process are depicted in Figure 4. The overall energy of the system stabilizes over time, oscillating within a narrow range around a consistent value, indicating that the cell models achieved full relaxation and attained a stable structure. The simulation utilized a step size of 1.0 fs, with system data recorded at intervals of 1000 steps. Temperature and pressure were regulated using the Anderson-Berendsen method. The electronic potential of Ewald was employed, and the Ewald force was statistically analyzed using the group-based method.

### 3.4. Model Reliability Verification

**Figure 5:**
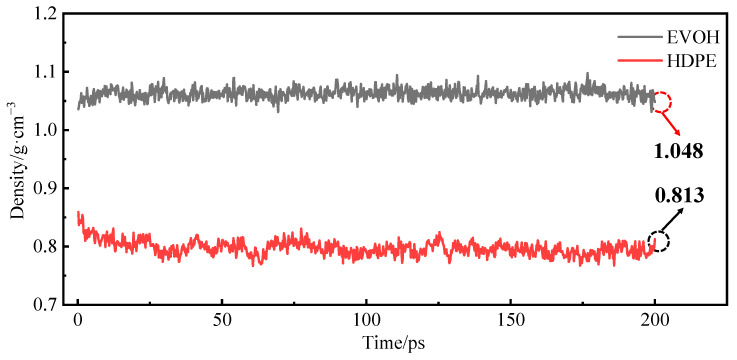
The densities of HDPE and EVOH during the relaxation process.

During the simulation, the HDPE and EVOH models exhibit fully amorphous structures. The density variations observed throughout the relaxation process are shown in Figure 5. It can be seen that the final density of HDPE is determined to be 0.813 g/cm^3^, a value close to the 0.805 g/cm^3^ reported by Dutta [27] and the 0.795 g/cm^3^ reported by Zheng [20] in their respective simulations. In comparison to the actual density of 0.855 g/cm^3^ [28] for HDPE in an amorphous state, the relative error is calculated to be 4.91%. Similarly, for EVOH, the observed density of 1.048 g/cm^3^ results in a relative error of 4.73% when compared to the true density of 1.10 g/cm^3^ [29]. Consequently, the density discrepancies for the HDPE and EVOH cell models fall below 5%, indicating the validity of the models proposed in this study.

**Figure 6:**
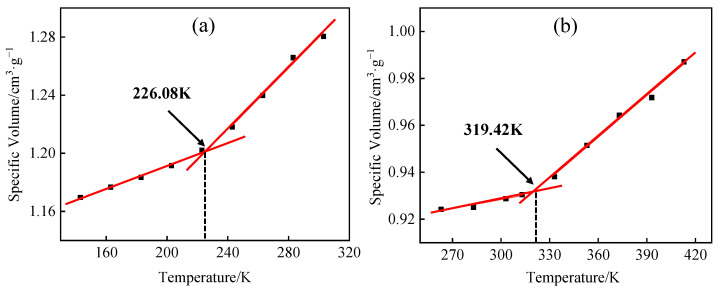
Glass transition temperature of (**a**) HDPE; (**b**) EVOH.

The glass transition is a commonly observed phenomenon in polymers, and the model’s accuracy can be further confirmed by the glass transition temperature. During a polymer’s glass transition, numerous physical characteristics undergo alterations, thus enabling the determination of the glass transition temperature by assessing variations in these physical attributes, such as specific volume. The HDPE and EVOH cells constructed were exposed to NVT + NPT treatment within the temperature ranges of 143–303 K and 263–413 K, respectively. The specific volume–temperature relationship was determined and is shown in Figure 6. The glass transition temperatures of HDPE and EVOH were found to be 226.08 K and 319.42 K, respectively, aligning with the experimental findings [30,31]. In conclusion, the model developed in this study is considered reliable.

In order to improve the validation of the simulation technique and its results, the permeability coefficient of a thin film made of HDPE was evaluated using a VAC-V2 pressure differential gas permeameter at a temperature of 30 °C. The experiment involved applying a pressure differential of 0.1 MPa across the sample, monitoring the pressure alteration on the low-pressure side resulting from gas permeation through the thin film, and determining the permeability of hydrogen. The permeability coefficient of hydrogen was determined to be 1.52 × 10^−13^ cm^3^ (STP) cm·cm^−2^·s^−1^·Pa^−1^. Conversely, the simulated value under identical conditions (30 °C, 0.1 MPa) was calculated to be 1.37 × 10^−12^ cm^3^ (STP) ·cm·cm^−2^·s^−1^·Pa^−1^. The HDPE film sample utilized in the experimental analysis exhibited a semi-crystalline nature with a crystallinity level of 57.8%, whereas the HDPE model used in the simulation was entirely amorphous. Given that gas permeation primarily occurs within the amorphous segment of the polymer, the conversion of gas molecule solubility, diffusion, and the permeability coefficient between the amorphous and actual semi-crystalline polymer can be facilitated through Equations (7) –(9) [20,32,33,34].

(7) $$S_{a}=S_{s}/\theta_{a}$$

(8) $$D_{a}=3D_{s}/2\theta_{a}$$

(9) $$P_{a}=3P_{s}/2\theta_{a}^{2}$$

where subscript *s* denotes that the polymer is completely amorphous; subscript *a* denotes that the polymer is semi-crystalline; and *θ_a_* is the volume fraction of the amorphous region, %.

The permeability of fully amorphous HDPE was analyzed using the formula above, resulting in a permeability coefficient of 1.63 × 10^−13^ cm^3^ (STP)·cm·cm^−2^·s^−1^·Pa^−1^ for HDPE with a crystallinity level of 57.8%. The simulation findings closely align with experimental data, demonstrating a high level of concordance with only a 7.24% margin of error. The disparity observed between the simulated and experimental results may be attributed to the varying scales of analysis employed, with the simulation concentrating on a microscopic level and the experimental assessment conducted at a macroscopic level, possibly overlooking certain external factors. In general, the simulated results in this investigation exhibit close correspondence with the experimental observations in terms of the order of magnitude, indicating the dependability of the molecular dynamics (MD) outcomes delineated.

## 4. Results and Discussion

### 4.1. Solubility Coefficient

**Figure 7:**
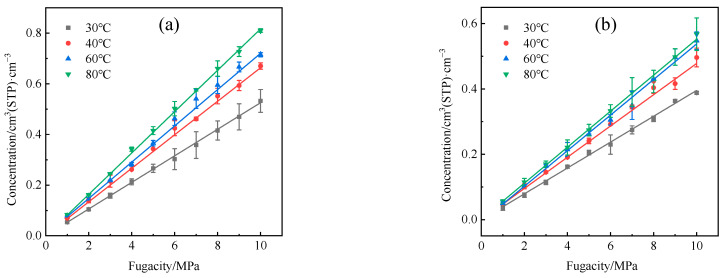
Adsorption isotherm of hydrogen: (**a**) HDPE; (**b**) EVOH.

**Figure 8:**
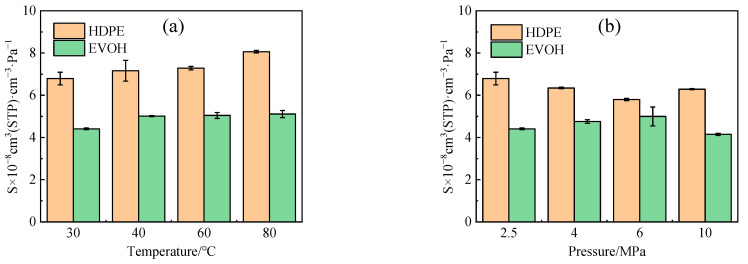
Solubility coefficients of hydrogen in HDPE and EVOH: (**a**) 2.5 MPa; (**b**) 30 °C.

The hydrogen adsorption isotherms at various temperatures were obtained after the adsorption, as illustrated in Figure 7. A nearly linear correlation between hydrogen adsorption capacity and time was observed, consistent with previous research by He et al. [35]. Ideally, the adsorption behavior of hydrogen and similar low-molecular-weight gases can be elucidated using Henry’s law [36], wherein the solubility coefficient is indicated by the limiting slope of the adsorption isotherm as the fugacity tends towards zero. According to Equation (3), the solubility coefficients of H_2_ in HDPE and EVOH in different conditions were calculated and are shown in Figure 8. The solubility coefficient of H_2_ in EVOH is significantly lower than that in HDPE, primarily attributed to the distinct structural characteristics of the two materials. EVOH, a copolymer of ethylene and vinyl alcohols, has a more complex molecular structure compared to HDPE. The molecular side chains of EVOH exhibit increased resistance to the permeation of hydrogen molecules, thus hindering the dissolution of H_2_ within EVOH.

The data presented in Figure 8a illustrate that the solubility coefficient of H_2_ exhibited increases of 18.7% and 15.9% for HDPE and EVOH, respectively, as the temperature rose from 30 °C to 80 °C. This observed phenomenon deviates from the conventional pattern of decreasing solubility coefficients as temperatures increase. This deviation is characterized by a phenomenon known as “reverse dissolution” [15,37,38], wherein gases with low critical temperatures, such as hydrogen, helium, nitrogen, and oxygen, exhibit increased solubility in the polymer as temperatures rise. This atypical behavior occurs because it is difficult to dissolve gases with low critical temperatures into polymers at low temperatures. However, the free volume in polymers increases with an increase in temperature, which increases the chance for gases with low critical temperatures to dissolve into polymers [20].

It can be seen from Figure 8b that the solubility coefficient of H_2_ in HDPE and EVOH exhibits a reverse trend with pressure. With increasing pressure, the solubility coefficient of H_2_ in HDPE gradually declines from 6.8 × 10^−8^ cm^3^ (STP)·cm^−3^·Pa^−1^ to 5.8 × 10^−8^ cm^3^ (STP)·cm^−3^·Pa^−1^, before experiencing a slight rise to 6.3 × 10^−8^ cm^3^ (STP)·cm^−3^·Pa^−1^. This change represents a 7.4% variation. Conversely, the solubility coefficient of H_2_ in EVOH increases from 4.4 × 10^−8^ cm^3^ (STP)·cm^−3^·Pa^−1^, reaching a peak at 5.0 × 10^−8^ cm^3^ (STP)·cm^−3^·Pa^−1^ at 6 MPa, and then, decreases to 4.1 × 10^−8^ cm^3^ (STP)·cm^−3^·Pa^−1^ at 10 MPa, reflecting a change rate of 6.8%. Overall, the impact of pressure on the solubility coefficient of hydrogen is constrained, aligning with findings reported in prior research [39].

**Figure 9:**
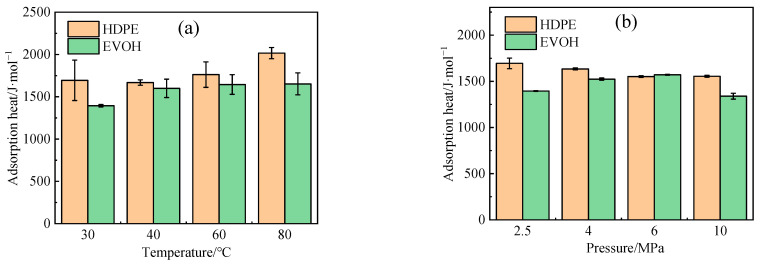
The isosteric heat of H_2_ adsorption in HDPE and EVOH: (**a**) 2.5 MPa; (**b**) 30 °C.

During the adsorption process, gas molecules migrate toward the solid surface, leading to a reduction in molecular velocity and the release of heat. The isosteric heat of adsorption (*Q_st_*) serves as a measure of the strength of the interaction between the adsorbate and adsorbent molecules. Typically, a higher isosteric heat of adsorption indicates a greater adsorption capacity and an increased solubility coefficient. By utilizing the Clausius–Clapeyron equation [40] (Equation (10)), the isosteric heat of adsorption (*Q_st_*) for hydrogen in HDPE and EVOH can be determined under various conditions, as illustrated in Figure 9.

(10) $$Q_{st}=-RT^{2}\left(\frac{\partial lnP}{\partial T}\right)n_{a}$$

where *Q_st_* is the isosteric heat of adsorption, J·mol^−1^; *R* is the universal gas constant, 8.31451 J·mol^−1^·K^−1^; *P* is the adsorption pressure, Pa; *T* is the simulated temperature, K; and *n_a_* is the amount of adsorption, mol·g^−1^.

With rising temperatures, the isosteric heat of adsorption experiences an increase (Figure 9a), leading to heightened molecular motion and subsequently enhancing the adsorption capacity. Concurrently, as pressure levels rise, the range of isosteric heat of adsorption in the two materials (Figure 9b) exhibits a similarity to the alteration observed in the dissolution coefficient illustrated in Figure 8b. Nevertheless, as illustrated in Figure 9b, the isosteric heat of H_2_ adsorption in HDPE and EVOH demonstrates nearly identical values at 6 MPa, whereas the solubility coefficient of H_2_ in these materials shows a significant disparity. This discrepancy can be attributed to the adsorption process being influenced by two key factors: the entropy alteration during solvation, as indicated by the adsorption heat, and the equilibrium of forces between polymer and gas molecules. Hydrogen, as a non-polar gas, lacks specific interactions with the polymeric main chain. In contrast, the side-chain hydroxyl group of EVOH establishes hydrogen bonds that enhance cohesion, thereby restricting the mobility of polymer segments and impeding the permeation of hydrogen through the polymer matrix.

### 4.2. Diffusion Coefficient

**Figure 10:**
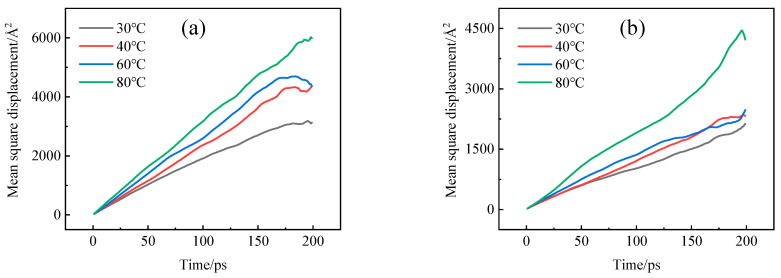
MSD curves of hydrogen molecules in (**a**) HDPE and (**b**) EVOH.

**Figure 11:**
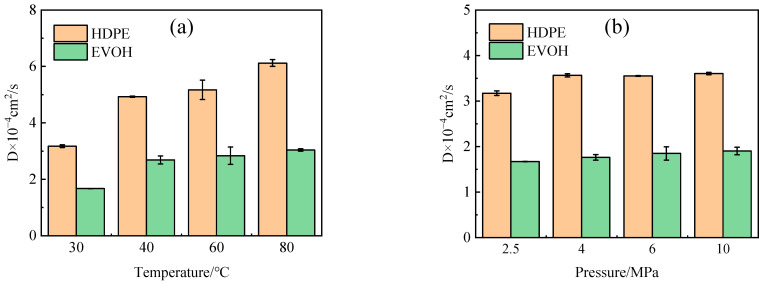
Diffusion coefficient of hydrogen in HDPE and EVOH: (**a**) 2.5 MPa; (**b**) 30 °C.

The Forcite module simulated the diffusion process after the dynamic relaxation of diffused cells. MSD curves illustrating the diffusion of hydrogen in HDPE and EVOH at different temperatures were plotted, as shown in Figure 10. The results demonstrated a linear correlation between the Mean Squared Displacement (MSD) and time in all experimental conditions. By applying Einstein’s law (Equations (4) and (5)), the diffusion coefficients of H_2_ in HDPE and EVOH were calculated, as illustrated in Figure 11. It was observed that the diffusion coefficient of hydrogen in both materials exhibited an increase with rising temperature and pressure. Specifically, with the temperature rising from 30 °C to 80 °C, the diffusion coefficients of hydrogen in HDPE and EVOH increased by 92.9% and 81.6%, respectively. However, the influence of pressure on the diffusion of hydrogen was found to be negligible. Beyond a pressure threshold of 4 MPa, the diffusion coefficients of hydrogen in HDPE and EVOH exhibited a consistent level.

**Figure 12:**
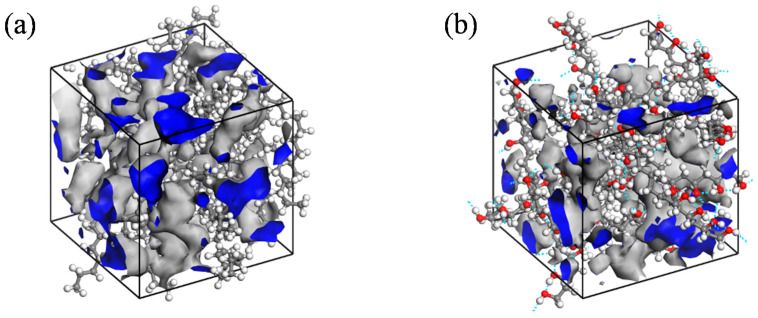
Free volume distribution of (**a**) H_2_/HDPE and (**b**) H_2_/EVOH.

The process of gas molecule diffusion in polymers is frequently explained using the free volume theory [41]. This theory suggests that the polymer volume can be divided into two distinct components: the volume taken up by the polymer chain, and the interstitial space between the molecular chains, referred to as the free volume. This free volume facilitates molecular movement by allowing molecules to modify their configuration through rotational and translational movements, thus making space for their activities. A larger proportion of free volume results in an increased surface area for gas molecules to move through. The free volume of the model was quantified using the Atom Volume and Surface module, as depicted in the shaded region in Figure 12.

**Figure 13:**
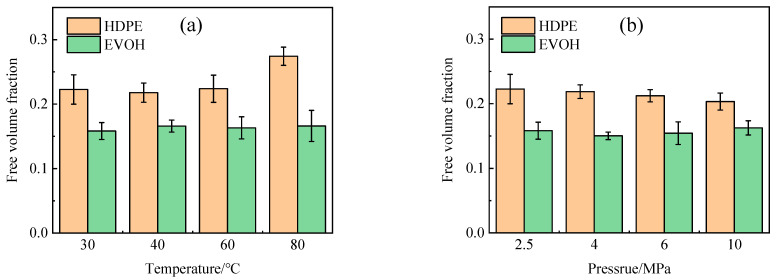
Free volume fraction of hydrogen in HDPE and EVOH: (**a**) 2.5 MPa; (**b**) 30 °C.

The free volume fraction represents the proportion of unoccupied space within the total volume, as illustrated in Figure 13. It is evident from the data presented in Figure 13a that the free volume fraction escalates as the temperature rises. This phenomenon occurs due to the expansion of the polymer volume with increasing temperature, resulting in a higher amount of free volume. Consequently, this increase in free volume facilitates the diffusion process and increases the diffusion coefficient. Moreover, elevated temperatures enhance the thermal motion of small gas molecules, thereby aiding in the displacement of molecules from confined spaces, thus facilitating the diffusion process. Conversely, changes in pressure do not significantly impact the free volume fraction, aligning with the observations on the diffusion coefficient, suggesting that the effect of pressure on the hydrogen diffusion coefficient can be considered negligible. Significantly, the diffusion coefficient of hydrogen within HDPE is approximately twice as large as that within EVOH, as illustrated in Figure 11. This can be attributed to the distinctive chain segment structure present in EVOH, characterized by the presence of hydroxyl groups within the molecule. The strong hydrogen bonding among EVOH molecules enhances their cohesive interactions, leading to a higher degree of molecular chain stacking and a reduced free volume fraction. Consequently, the hindered diffusion of H_2_ within EVOH compared to HDPE can be attributed to these structural differences [42].

### 4.3. Permeability Coefficient

**Figure 14:**
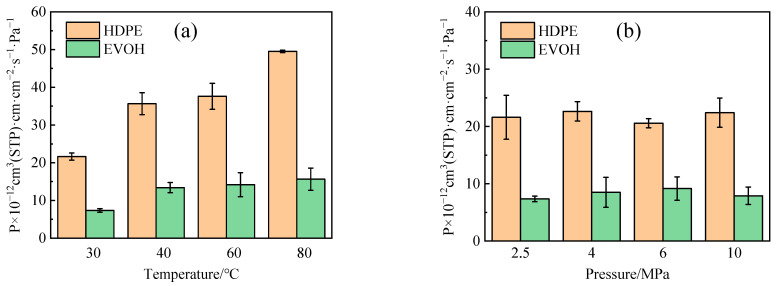
Permeability coefficient of hydrogen in HDPE and EVOH: (**a**) 2.5 MPa; (**b**) 30 °C.

Based on the adsorption-diffusion theory and Equation (1), the permeability coefficients of H_2_ in HDPE and EVOH were calculated under various conditions, as illustrated in Figure 14. The permeability coefficient of H_2_ in HDPE surpasses that in EVOH, aligning with the observed trend in the diffusion and solubility coefficients. The permeability coefficient of H_2_ in both HDPE and EVOH exhibits a notable increase from 30 °C to 80 °C, with growth rates of 129.0% and 112.7%, respectively (Figure 14a). In contrast to the impact of temperature on the permeability coefficient, the influence of pressure is deemed insignificant (Figure 14a), consistent with the results in the literature [20,43]. The variations in the permeability coefficient of hydrogen in HDPE and EVOH, ranging from 2.5 MPa to 10 MPa, are recorded at 3.7% and 7.5%, respectively. In low-pressure settings (below 10 MPa) with gases that are somewhat condensable, gas permeability typically relies solely on temperature rather than the gas concentration within the polymer or the hydrostatic pressure exerted on the polymer membrane [44]. In other words, in environments characterized by low hydrogen pressure, changes in pressure levels do not impact the material’s permeability.

Moreover, the order of magnitude of the diffusion coefficient (Figure 11) surpasses that of the solubility coefficient (Figure 8). The adsorption mechanism of gas within a polymer entails intricate interactions between polymer and gas molecules [45], a phenomenon dictated by the equilibrium of intermolecular forces between the polymer and gas, as well as the entropy alteration during the solution process. Diffusion, on the other hand, is a purely physical occurrence where gas molecules traverse through polymer chains within available free space, with minimal to negligible interaction between the polymer and gas molecules. Consequently, the primary determinant of hydrogen permeability at a specific temperature is the variation in diffusion (physical movement) rather than the impact of adsorption (molecular interaction).

### 4.4. Permeability Mechanism

**Figure 15:**
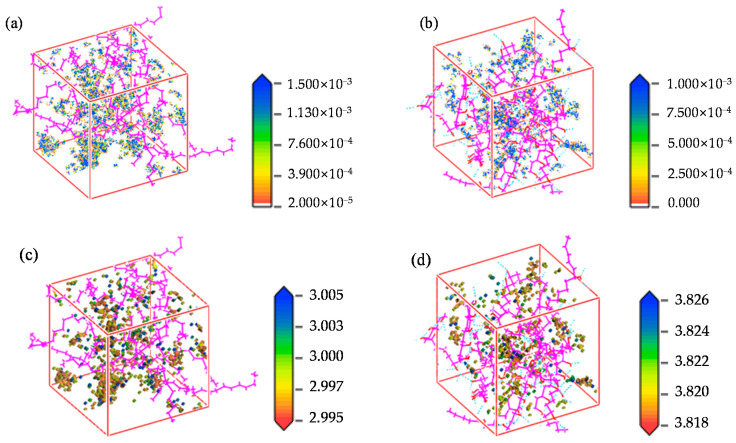
Density field distribution of hydrogen ((**a**) HDPE, (**b**) EVOH) and isopycnic ((**c**) HDPE, (**d**) EVOH).

**Figure 16:**
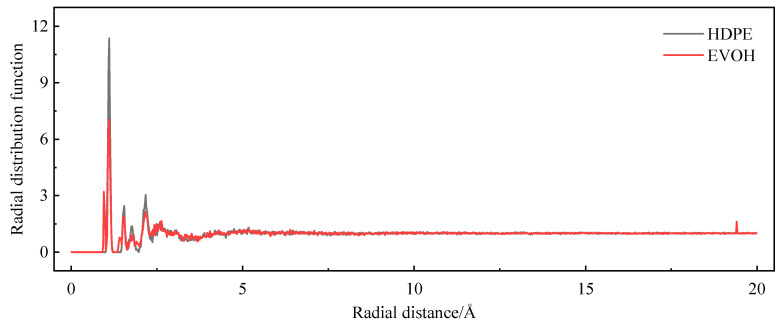
Radial distribution function of hydrogen.

Based on the adsorption module, a density field distribution diagram and an isodensity distribution diagram of hydrogen can be derived from the cell after adsorption, as illustrated in Figure 15. The adsorption density field distribution of hydrogen indicates the concentrated distribution area of hydrogen molecules in the cell. Figure 15a,b demonstrate that the adsorption sites of hydrogen are not uniformly distributed, but rather, clustered within both cells. Nevertheless, owing to the presence of hydrogen bonds in EVOH, the adsorption of hydrogen is lower in comparison to HDPE, with the majority of hydrogen being concentrated in regions devoid of hydrogen bonds. To more clearly reflect the strength of the interaction energy between hydrogen and polymers, the radial distribution function of hydrogen in the two materials was analyzed using the Forcite module, as shown in Figure 16. The radial distribution function of hydrogen in both materials displays similar shape characteristics with a prominent peak value. The peak value of hydrogen in HDPE surpasses that in EVOH, indicating a more robust interaction between hydrogen and HDPE. The isodensity distributions are shown in Figure 15c,d, where regions of high energy are denoted in blue and low-energy areas in red. It can be seen that hydrogen molecules tend to cluster in low-energy regions, specifically the red sections in Figure 15c,d, aligning with the high-density hydrogen concentration area (blue section) in Figure 15a,b. In summary, the absorption of hydrogen in HDPE and EVOH predominantly occurs in low-energy regions, signifying aggregative adsorption.

**Figure 17:**
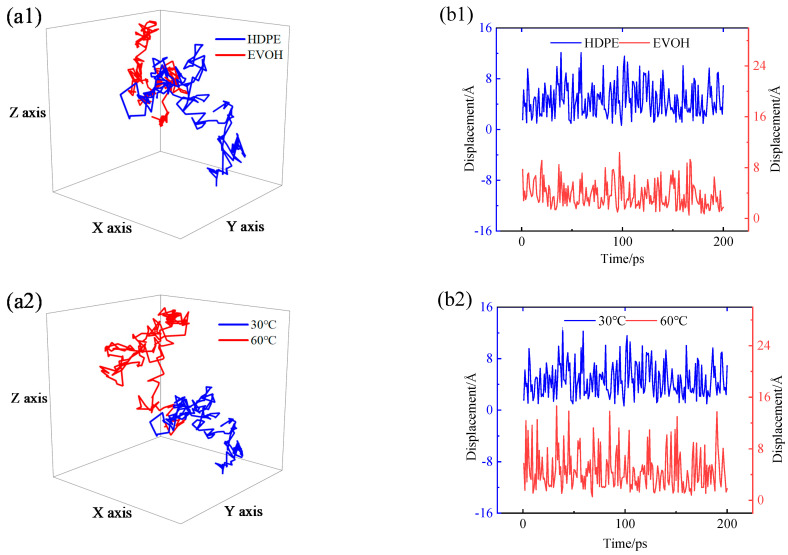

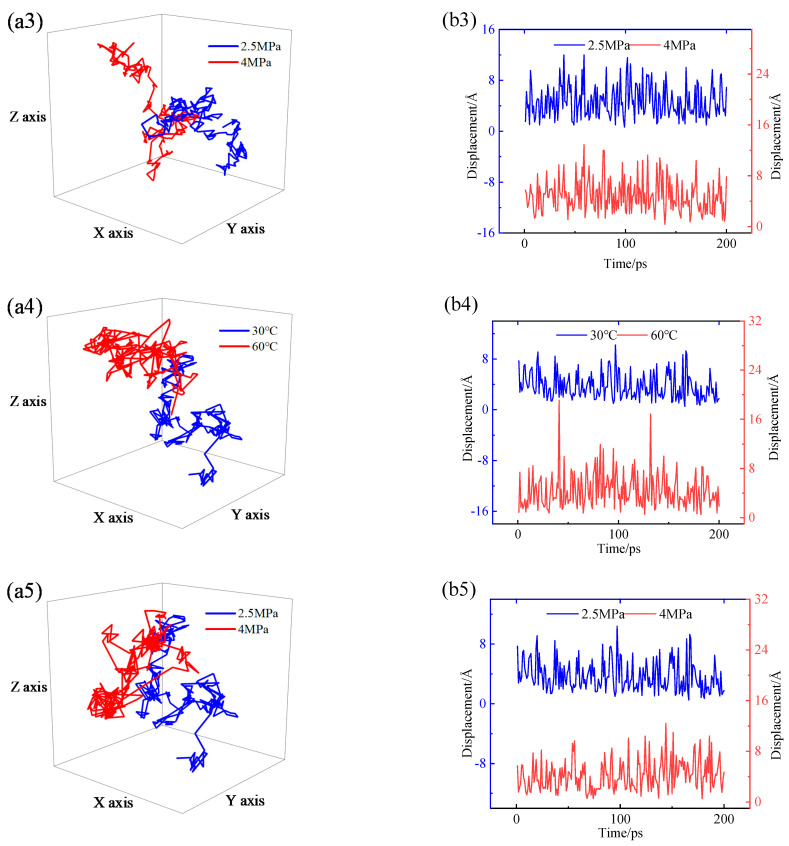
(a) Three-dimensional diffusion trajectory and (b) displacement of H_2_: (**a1**,**b1**) 30 °C, 2.5 MPa; (**a2**,**b2**) HDPE, 2.5 MPa; (**a3**,**b3**) HDPE, 30 °C; (**a4**,**b4**) EVOH, 2.5 MPa; (**a5**,**b5**) EVOH, 30 °C.

To visually observe and analyze the diffusion behavior of H_2_ within HDPE and EVOH, a script was utilized to extract the trajectory of H_2_ molecules post-simulated diffusion from the trajectory file. Subsequent processing facilitated the acquisition of the three-dimensional diffusion trajectories and displacement patterns of H_2_ under varying conditions, as illustrated in Figure 17. The analysis revealed that at 30 °C and 2.5 MPa, hydrogen molecules exhibit more pronounced movement within HDPE compared to EVOH, predominantly vibrating within holes with an approximate amplitude of 0.4 nm, as depicted in Figure 17(a1,b1). Notably, a few substantial transitions between holes were also observed, with amplitudes reaching around 0.8 nm, as shown in Figure 17(b1). With increasing temperature, the range of motion of hydrogen molecules expanded, progressing from around 0.4 nm at 30 °C to 0.6 nm at 60 °C. Moreover, the number of hydrogen transitions between holes increased from about 11 at 30 °C to 22 at 60 °C in HDPE, and from about 4 at 30 °C to 10 at 60 °C in EVOH (transitions exceeding 0.6 nm were considered jumps between holes), indicating a heightened diffusion capacity, as illustrated in Figure 17(a2,b2,a4,b4). Particularly noteworthy is the presence of two substantial jumps of approximately 1.6 nm at 60 °C, as depicted in Figure 17(b4). Conversely, when pressure variations were introduced while maintaining a constant temperature, minimal impact on the molecular motion of hydrogen was observed, with transition distances remaining around 0.6 nm, as shown in Figure 17(a3,b3,a5,b5), aligning with the aforementioned observations. The diffusion mechanism of gas molecules within polymers, as delineated in Figure 17, is supported by existing literature findings and conclusions. Notably, the distinction lies in the smaller diameter of hydrogen molecules, longer duration of inter-hole transitions, and stronger diffusion capability compared to methane and oxygen molecules [46].

According to the aforementioned analysis, it can be inferred that the permeability mechanisms of hydrogen in HDPE and EVOH exhibit similarities. The mechanisms involve an aggregation adsorption process in the low-potential-energy region and a diffusion process characterized by vibration and inter-hole transitions within the material. The permeability of hydrogen in both HDPE and EVOH is predominantly influenced by temperature rather than pressure. Additionally, EVOH demonstrates superior hydrogen barrier properties in comparison to HDPE.

## 5. Conclusions

1. The adsorption heat and free volume of hydrogen (H_2_) in high-density polyethylene (HDPE) and ethylene–vinyl alcohol (EVOH) exhibit an increasing trend with rising temperatures, leading to corresponding changes in solubility, diffusion, and permeability coefficients. Specifically, as the temperature increases from 30 °C to 80 °C, the solubility, diffusion, and permeability coefficients of hydrogen in HDPE experience increments of 18.7%, 92.9%, and 129.0%, respectively. In the case of EVOH, the values are 15.9%, 81.6%, and 112.7%. Conversely, the diffusion, solubility, and permeability coefficients of hydrogen in HDPE and EVOH are almost unaffected under low-pressure conditions in the study pressure range (2.5–10 MPa).
2. Due to the unique chain segment configuration of EVOH, a robust hydrogen bond is established among the hydroxyl groups, impeding the penetration of hydrogen. Consequently, EVOH exhibits superior hydrogen barrier characteristics compared to HDPE, as evidenced by the significantly lower diffusion, solubility, and permeability coefficients of hydrogen within EVOH.
3. The permeability mechanisms of hydrogen (H_2_) in HDPE and EVOH exhibit similarities, involving an aggregation adsorption process occurring within a low potential energy range and a diffusion process characterized by vibration-induced transitions between holes. During adsorption, hydrogen molecules tend to accumulate in regions of low energy, with EVOH specifically showing a preference for areas lacking hydrogen bonds. During the subsequent diffusion phase, hydrogen molecules exhibit vibrational motion within a hole over a limited range before transitioning to another hole at a greater distance. The hydrogen molecules then vibrate in their new position. Elevated temperatures lead to increases in the motion range and transition frequency of hydrogen molecules.
4. The affinity of hydrogen with HDPE is more pronounced compared to that with EVOH, leading to an increased number of adsorption sites and enhanced hydrogen adsorption capacity in HDPE. Hydrogen molecules exhibit higher mobility within HDPE as opposed to EVOH, characterized by greater hole amplitude and a higher number of transitional holes during the diffusion phase. Consequently, EVOH demonstrates superior hydrogen barrier characteristics when compared to HDPE.

## Data Availability

Data are contained within the article.
